# Pedaling rate is an important determinant of human oxygen uptake during exercise on the cycle ergometer

**DOI:** 10.14814/phy2.12500

**Published:** 2015-09-14

**Authors:** Federico Formenti, Alberto E Minetti, Fabio Borrani

**Affiliations:** 1 Nuffield Department of Clinical Neurosciences, Nuffield Division of Anaesthetics, John Radcliffe Hospital, University of OxfordOxford, UK; 2 Department of Sport and Exercise Science, The University of AucklandAuckland, New Zealand; 3 Faculty of Medicine, Department of Pathophysiology and Transplantation, University of MilanMilan, Italy; 4 Institute of Sport Sciences, Department of Physiology, Faculty of Biology and Medicine, University of LausanneLausanne, Switzerland

**Keywords:** Biomechanics, exercise physiology, oxygen uptake

## Abstract

Estimation of human oxygen uptake (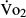) during exercise is often used as an alternative when its direct measurement is not feasible. The American College of Sports Medicine (ACSM) suggests estimating human 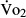 during exercise on a cycle ergometer through an equation that considers individual's body mass and external work rate, but not pedaling rate (PR). We hypothesized that including PR in the ACSM equation would improve its 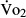 prediction accuracy. Ten healthy male participants’ (age 19–48 years) were recruited and their steady-state 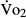 was recorded on a cycle ergometer for 16 combinations of external work rates (0, 50, 100, and 150 W) and PR (50, 70, 90, and 110 revolutions per minute). 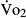 was calculated by means of a new equation, and by the ACSM equation for comparison. Kinematic data were collected by means of an infrared 3-D motion analysis system in order to explore the mechanical determinants of 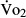. Including PR in the ACSM equation improved the accuracy for prediction of sub-maximal 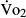 during exercise (mean bias 1.9 vs. 3.3 mL O_2_ kg^−1^ min^−1^) but it did not affect the accuracy for prediction of maximal 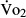 (*P *>* *0.05). Confirming the validity of this new equation, the results were replicated for data reported in the literature in 51 participants. We conclude that PR is an important determinant of human 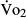 during cycling exercise, and it should be considered when predicting oxygen consumption.

## Introduction

Human metabolism and its changes induced by physical exercise have been of great interest to physiologists for over a century (Douglas et al. 1913). Appreciation of the relationship between the level of physical exercise and metabolism is an important factor for our understanding of human physiology, with implications for medicine, nutrition, and especially for exercise testing and prescription (Garber et al. 2011). In particular, the oxygen uptake (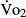) during aerobic exercise is one of the most important references for exercise prescription. Several noninvasive technologies are available to accurately measure gas exchange at the mouth [e.g., (McLaughlin et al. 2001; Larsson et al. 2004)] from the resting state to maximal exercise levels. While being employed in research, clinical and elite level sports’ settings, these technologies are rarely used with the general public due to a variety of factors, including equipment costs and skills required for their use.

When it is not possible to measure gas exchange at the mouth, theoretical modeling affords the prediction of metabolic responses to exercise. Prediction of 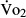 at sub-maximal exercise level through theoretical models is not as accurate as direct 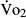 measurement, but it offers several advantages like reduced costs, and capacity to estimate maximal 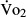 (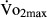) in populations where direct assessment of individuals’ maximal exercise aerobic power may not be feasible [e.g., patients with heart failure (Noonan and Dean 2000; Balady et al. 2010)], or where equipment or expertise to measure 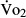 are not available.

Several equations have been proposed for the prediction of 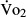 during exercise, in particular for exercise on the cycle ergometer (Åstrand and Ryhming 1954; McCole et al. 1990; Lang et al. 1992; Latin et al. 1993; Francescato et al. 1995; Londeree et al. 1997; Stanforth et al. 1999; Emanuele and Denoth 2012). These equations can be rather accurate, yet complex, taking into account several determinants of 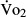, for example individual's mass, gender, age, work rate, and pedaling rate (PR). A trade-off exists between a 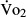 prediction equation's complexity and its potential accuracy, which is not necessarily improved by a greater number of variables. Complex equations are suitable for research or particular settings (e.g., elite athletes), while simpler ones, with their limitations, appear to be more useful in the practitioner's daily application.

The American College of Sports Medicine (ACSM) recommends the use of a straightforward 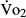 prediction equation for exercise on the cycle ergometer (ACSM, 2009). It is generally accepted that this equation is accurate for work rates between 50 and 200 W. In this equation, determinants of mass-specific 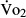 (mL O_2_ kg^−1^ min^−1^) are individual's mass and work rate:

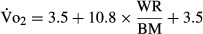
1

Here, the first 3.5 mL O_2_ kg^−1^ min^−1^ is an assumed constant value for resting 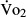, BM indicates participant's mass (kg), the second 3.5 mL O_2_ kg^−1^ min^−1^ is an assumed 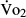 constant value for unloaded pedaling (i.e., at 0 W, regardless of PR), and WR indicates external work rate (Watts). In this equation WR is equal to the product of resistance (N), distance per revolution (m) and PR (Hz), so eq. (1) overlooks the relative contribution of PR in determining WR. For example, given a 6 m distance per pedal revolution, whether an individual pedals at a resistance of about 53 N and cadence of 0.5 Hz (30 revolutions per minute, RPM), or at a resistance of about 13 N and cadence of 2.0 Hz (120 RPM), the equation would present exactly the same WR of 160 W, whereas the two physiological responses to exercise would be very different.

Pedaling rate is a strong determinant of 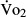 during exercise on the cycle ergometer: from an optimal PR, 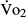 increases with PR when pedaling against no resistance (Seabury et al. 1977), or against a resistance (Zoladz et al. 2000). We hypothesized that prediction of 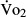 during exercise on the cycle ergometer through a new equation can be improved by considering PR as a determinant of 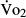, and that the inclusion of PR as a determinant of 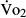 would not impair the ACSM equation's capacity to predict 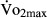. Furthermore, we explored the biomechanical determinants of 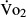 by means of kinematic data analysis.

## Methods

### Participants

Ten healthy male participants took part in the study (Table 1). Participants’ age, height, body mass and amount of physical exercise taken per week spanned across a rather wide range (19–48 years; 166–187 cm; 60–94 kg; 0–8 h of exercise per week). All participants were informed about the aims, procedure, and details of the study, and signed a consent form before taking part in the experiments. The studies conformed to the Declaration of Helsinki and had been approved by the local ethics committee.

**Table 1:** Participants’ individual and group characteristics

ID	Age (years)	Height (cm)	Weight (kg)	Exercise per week (hours)	V̇O2_max_ (mlO_2_ kg^−1^ min^−1^)	Maximal work rate (Watt)
1	35	179	80	5	52	400
2	20	184	60	7	51	293
3	19	166	77	5	41	303
7	22	175	79	3	41	336
9	48	170	68	5	41	337
10	21	181	94	0	50	382
11	26	185	71	8	51	321
14	21	187	74	7	*	*
15	21	183	78	3	60	376
16	23	172	65	5	41	283
Mean	26	178	75	5	48	337
SD	9	7	9	2	7	42

Participant ID number, age, height, weight, amount of physical exercise taken per week, 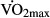, and maximal work rate achieved during the incremental test to exhaustion on the cycle ergometer.

* Participant ID 14 did not complete the 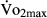
test.

### Protocol

Participants were asked to avoid vigorous exercise and alcohol and caffeine consumption for 48 h prior to the experiments; they were also asked to eat a light meal on the day of the experiments (i.e., light breakfast or lunch for experiments in the morning or afternoon respectively). Room temperature was kept at approximately 21°C throughout the experiments.

Participants visited the laboratory on three separate occasions, with at least 2 days rest between visits. Cycling position was chosen by the participants to meet individuals’ comfort requirements, with the knee flexed at approximately 30 degrees at the bottom of the pedal stroke. 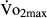 on a cycle ergometer was measured on the first visit. On the second and third visits 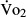 was measured on the cycle ergometer either at 50 and 90 revolutions per minute, or at 70 and 110 revolutions per minute; the sequence of pedaling rates (PR) was randomized between participants. Participants rested for a period of at least 40 min between two tests. Each PR was tested at four subsequent work rates (0, 50, 100, 150 W).

Pedaling and work rates were monitored on a computer-controlled, electrically braked cycle ergometer (Velotron, RacerMate, WA), which was calibrated using a dynamic calibration rig before the experiments (Abbiss et al. 2009). Heart rate was monitored by means of a heart rate monitor (Polar RS800CX, Polar Electro OY, Kempele, Finland).

During the course of each experiment, participants breathed through a face mask; 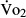 and 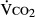 were recorded breath-by-breath through a portable gas analysis system (MetaMax 3B, CORTEX Biophysik GmbH, Leipzig, Germany); the accuracy and reliability of this system has been presented elsewhere (Macfarlane and Wong 2012). The turbine transducer was calibrated using a 3-liter syringe (Hans Rudolph, Kansas City, MO), and measured inspired and expired gas flow. The electrochemical oxygen analyzer and the infrared carbon dioxide analyzer were calibrated through gases of known concentration before each experiment (Larsson et al. 2004), and used to measure inspired and expired gases concentrations.

### Maximal oxygen uptake measurement on the cycle ergometer


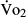
 was measured while participants rested on the cycle ergometer for 3 min. Participants were then asked to pedal at a self-selected PR (˜ 70 revolutions per minute) at 60 W for 6 min, followed by a continuous ramped increase in work rate of 30 W min^−1^ until exhaustion. Exhaustion was defined as the time when participants could no longer maintain the chosen PR. 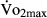 and maximal work rate were calculated as the average 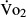 and work rate recorded in the last 30 sec of exercise.

### Oxygen uptake measurement on a cycle ergometer at different pedaling and work rates


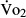
 was measured for 5 min at each combination of pedaling and work rates. The chain was disconnected from the cycle ergometer for the tests at 0 W, in order to reduce external work rate to a minimum. For each PR test, exercise started at 0 W for 5 min, followed by 50 W increments every 5 min, up to 150 W.

### Models for prediction of oxygen uptake

The prediction of 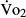 during exercise was initially performed through eq. (1), recommended by the ACSM (2009).

In addition, we developed a new equation following the theoretical approach of Minetti et al. (2001) and Minetti (2011). Our new equation considers that the mechanical internal work is caused by the kinetic energy of limbs with respect to the body center of mass (Fenn 1930). Moreover, since pedaling rate is proportional to the crank speed, and kinetic energy needs speed squared, it follows that work depends on PR raised to the power of two, and that work rate (= work/time = work x frequency) depends on PR^3^. In the quoted paper (Minetti 2011) 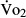 (Watts) has been modeled as the ratio between the total mechanical work rate (= external + internal work rate) and the efficiency of positive work (eff^+^):

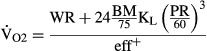
2

Here, WR indicates the external work rate (Watts), BM indicates participant's mass (kg), and PR indicates pedaling rate (revolutions per minute). The term accounting for the internal work rate is ‘weighed’ for the fractional mass of lower limbs within the body (= 24/75), multiplied by BM, by the inertia term K_L_ and, as mentioned, by the third power of the pedaling rate. In that study, a multiple nonlinear regression of this model on real data resulted in K_L_ = 0.469 and eff^+^ = 0.27. By using those coefficients, and by expressing eq. (2) in mass-specific terms (W kg^−1^), we obtain:

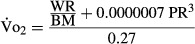
3

By further converting the metabolic units mL O_2_ kg^−1^ min^−1^, with 1 mL O_2_ = 20.5 J (corresponding to an average 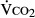/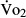 of 0.89), by adding 3.5 mL O_2_ kg^−1^ min^−1^ for resting metabolism and multiplying by 60, the resulting equation follows:

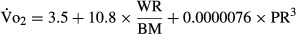
4where participant's mass, WR and PR [MWPR] are considered as variables.

In essence, this eq. (4) adds to eq. (1) a variable determinant of 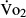 in the form of PR (revolutions per minute), which takes the place of the assumed 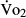 constant value for unloaded pedaling in eq. (1).


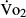
 values (measured or predicted) at different pedaling and imposed work rates were also used to estimate 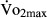, assuming (1) a linear relationship between heart rate and 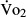, and (2) a maximal heart rate calculated as 220 minus participant's age in years (McArdle et al. 2010).

### Mechanical determinants of energetics for exercise on the cycle ergometer

We explored the biomechanics of exercise on the cycle ergometer through kinematic data collection using an eight-camera infrared 3-D motion analysis system (Vicon 612; Oxford Metrics Ltd, Oxford, UK) at a sampling frequency of 100 Hz. The spatial accuracy of the system is better than 1 mm (root mean square).

Participants were equipped with 18 infrared reflective markers’ (diameter 14 and 25 mm), attached to the skin overlying the following anatomical landmarks (symmetrically left and right sides): frontal part of the temporal bone, acromion, lateral humeral epicondyle, ulnar styloid process, greater trochanter, lateral femoral epicondyle, lateral malleolus, calcaneum, fifth metatarsal head.

Mechanical internal work has been obtained by calculating the (linear and rotational) speed of center of mass of lower limb segments, relative to the one of the whole body, and by summing the increases of their kinetic energies during the pedaling revolutions. Average mechanical internal work rate (P_int_, W/kg) is then computed by dividing the internal work by the duration of the sampled kinematics over an integral number of cycles.

### Prediction of oxygen uptake for previously published results

In order to test the validity of the MWPR equation, we compared 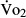 values for cycling exercise reported in the literature with values predicted by the MWPR and ACSM equations. We selected six published articles where participants’ 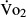 recorded at steady state, body mass, external work rate and PR were available (Gaesser and Brooks 1975; Seabury et al. 1977; Hagberg et al. 1981; Coast and Welch 1985; Francescato et al. 1995; Belli and Hintzy 2002). Overall, these studies allowed considering a total of 51 participants who were tested at different PR (range: 30–120 revolutions per minute), work rates (0–300 W), in different laboratories and years (1975–2002), and on different cycle ergometers.

### Statistical analysis

Pedaling and external work rates, and physiological variables recorded at steady state in the last 2 min of each stage of exercise were averaged and considered for further analysis. Differences between measured and predicted values were assessed statistically using repeated measures analysis of variance (ANOVA) (IBM SPSS Statistics for Windows, Version 20.0; Armonk, NY). Statistical significance was assumed at values of *P *<* *0.05. Agreement between measured and predicted 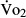 values with multiple observations per participant was assessed graphically with Bland–Altman plots (Bland and Altman 2007). Variables are presented as means ± SD, and 95% Confidence Intervals (CI), unless otherwise stated.

## Results

Table 1 shows the participants’ characteristics including age, height, mass, and number of hours of exercise taken per week. Table 1 also shows the results for 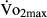 and maximal work rate recorded during the incremental exercise test to exhaustion on the cycle ergometer. Considering the protocol used for the incremental exercise test and the population studied, the values of 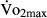 and maximal work rate recorded appeared normal.

### Including pedaling rate in the ACSM equation improved oxygen uptake prediction's accuracy during sub-maximal exercise on the cycle ergometer

**Figure 1:**
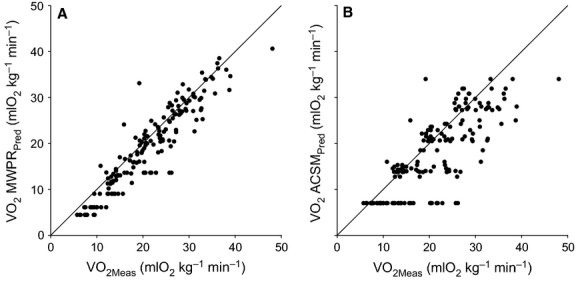
Submaximal 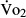 values predicted by (A) MWPR equation and (B) The American College of Sports Medicine (ACSM) equation are shown as a function of measured 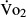. Each point shows the average 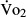 recorded over 2 min at steady state for one participant; each of the ten participants exercised at four power levels, each at four pedaling rates (*n* = 160). The straight line is the identity line. Meas, measured values. Pred, predicted values.

Figure 1 illustrates the 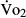 values predicted by the (A) MWPR and (B) ACSM equations plotted against 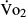 values measured during exercise on the cycle ergometer. Only 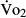, values predicted with the MWPR equation were not significantly different from the measured ones (*P *>* *0.05).

**Figure 2:**
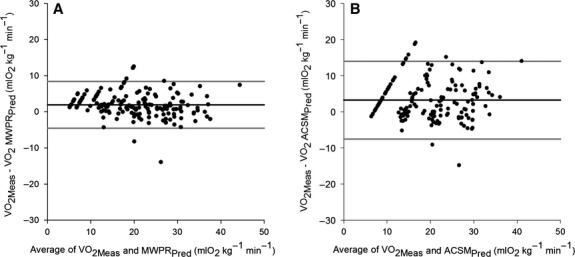
Bland–Altman plots show the degree of agreement between measured 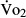 values, and 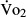 values calculated with (A) the MWPR and (B) the ACSM equations. The black line shows the bias, and the two gray lines show the bias ± 1.96 SD.

Figure 2 illustrates the degree of agreement between measured 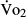 values and those predicted by the (A) MWPR and (B) ACSM equations. The bias for the 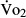 values predicted by the MWPR equation was 1.9 mL O_2_ kg^−1^ min^−1^ (SE ± 0.5), with limits of agreement between −4.6 (95% CI −6.5 to −3.4) and 8.6 (95% CI 7.4 to 10.5); the SD of the differences was 3.3 (SE ± 0.2).The within- and between-subject variances were respectively 9.2 (SE ± 1.1) and 2.0 (SE ± 1.2). The bias for the 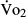 values predicted by the ACSM equation was 3.2 mL O_2_ kg^−1^ min^−1^ (SE ± 0.5), with limits of agreement between −7.7 (95% CI −9.6 to −6.3) and 14.1 (95% CI 12.7 to 16); the SD of the differences was 5.6 (SE ± 0.3). The within- and between-subject variances were, respectively, 30.3 (SE ± 3.5) and 0.7 (SE ± 1.2).

### Including pedaling rate (PR) in the ACSM equation did not affect the prediction of maximal oxygen uptake

**Figure 3:**
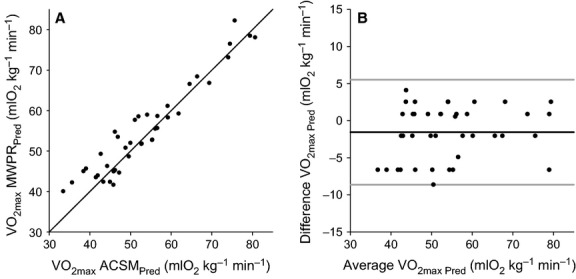
(A) Maximal 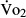 values predicted for our participants by the MWPR equation are shown as a function of maximal 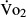 values predicted by the ACSM equation; each of the ten participants’ 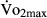 was calculated relative to four pedaling rates (*n* = 40). The straight line is the identity line. (B) The Bland–Altman plot shows the degree of agreement between 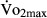 calculated with the MWPR and ACSM equations. The difference on the vertical axis is calculated as values calculated with the ACSM equation minus values calculated with the MWPR equation. The black line shows the bias, and the two gray lines show the bias ± 1.96 SD.

Figure 3A illustrates the 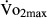 values predicted by the MWPR equation as a function of the 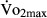
values predicted by the ACSM equation. These 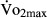 values predicted by the MWPR and ACSM equations were not significantly different (*P *=* *0.55). Measured 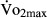 (48 ± 7 mL O_2_ kg^−1^ min^−1^; 95% C.I. 46–50) was not significantly smaller than the 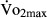 predicted by the MWPR equation (53 ± 10 mlO_2_ kg^−1^ min^−1^; 95% C.I. 50–56; *P *=* *0.13) and by the ACSM equation (51 ± 11 mL O_2_ kg^−1^ min^−1^; 95% C.I. 47–54; *P *=* *0.25). Figure 3B shows the agreement between the 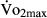 values predicted by the MWPR and ACSM equations: the bias for 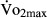 values was −1.6 mlO_2_ kg^−1^ min^−1^ (SE ± 0.2), with limits of agreement between −8.7 (95% CI −11.3 to −7.3) and 5.4 (95% CI 3.9–7.9). The SD of the differences was 3.6 (SE ± 0.5). The within- and between-subject variances were, respectively, 16.7 (SE ± 4.5) and −3.6 (SE ± 1.1).

### The MWPR equation improved the ACSM equation's prediction's accuracy for results reported in the literature

**Figure 4:**
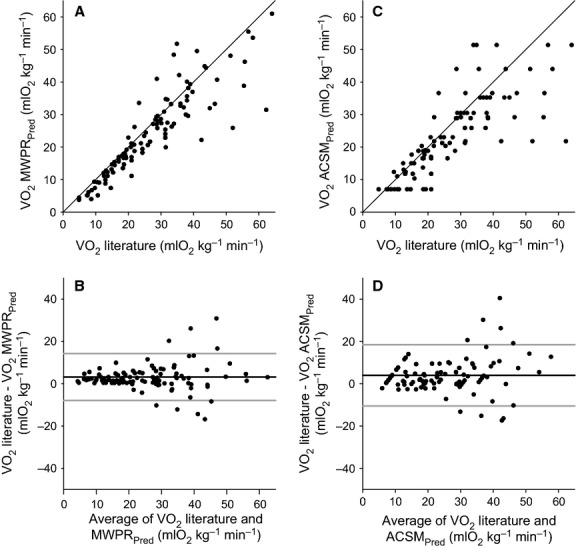
(A) 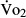 values predicted by MWPR equation are shown as a function of 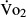 values reported in the literature for exercise on the cycle ergometer. Each point is the average of at least 2 min of steady-state exercise. The straight line is the identity line. (B) The Bland–Altman plot shows the degree of agreement between 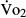 calculated with the MWPR equation and those reported in the literature. Similarly, (C) 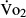 values predicted with the ACSM equation are plotted against 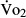 values reported in the literature; (D) the degree of agreement between 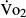 values calculated with the ACSM equation and those reported in the literature is illustrated in the Bland–Altman plot. In subpanels B and D, the horizontal black line shows the bias, and the two gray lines show the bias ± 1.96 SD.

Figure 4A illustrates 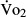 values predicted by MWPR equation as a function of the 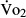 values for exercise on a cycle ergometer reported in the literature. 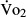 values predicted by the MWPR equation were very similar to the 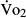 values reported in the literature, especially below 40 mL O_2_ kg^−1^ min^−1^, where results were very close to the identity line. Figure 4B illustrates the degree of agreement between 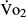 values reported in the literature and those predicted by the MWPR equation. The bias for the 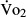 values predicted by the MWPR equation was 3.2 mL O_2_ kg^−1^ min^−1^ (SD ± 5.7; 95% CI 2.8–3.6), with limits of agreement between −7.9 (95% CI −8.6 to −7.1) and 14.3 (95% CI 13.6 to 15.1).

Figure 4C is similar to Figure 4A, but illustrates 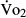 values predicted by the ACSM equation. 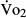 values predicted by the ACSM equation were not as close to the identity line as values predicted by the MWPR equation. Figure 4D illustrates this relatively smaller degree of agreement between 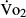 values reported in the literature and those predicted by the ACSM equation. The bias for the 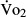 values predicted by the ACSM equation was 4.0 mL O_2_ kg^−1^ min^−1^ (SD ± 7.3; 95% CI 3.4–4.6), with limits of agreement between −10.5 (95% CI −11.5 to −9.6) and 18.5 (95% CI 17.6 to 19.5).

### Kinematic analysis supports the rationale for considering pedaling rate in the estimation of oxygen uptake on the cycle ergometer

Table 2 reports the average mechanical internal work rates for all the pedaling rates and the imposed external work rates. Internal work rate for a given pedaling rate increased little between 50 W and 150 W. In contrast, for a given external work rate, internal work rate increased almost 10 times with pedaling rate going from 50 to 110 RPM. The results were independent from the imposed external power load, as expected [eq. (3) in (Minetti et al. 2001)], thus all the data have been pooled as to obtain mean ± SD values for each pedaling rate (last two columns in Table 2).

**Table 2:** External and internal work rate values, associated with the corresponding pedaling rates

External work rate	50 W (0.68 W kg^−1^)		100 W (1.36 W kg^−1^)		150 W (2.04 W kg^−1^)		All	
Internal work rate	Mean	SD	Mean	SD	Mean	SD	Mean	SD
Pedaling Rate								
50	0.105	0.008	0.105	0.010	0.113	0.011	0.108	0.010
70	0.278	0.021	0.314	0.051	0.313	0.082	0.303	0.058
90	0.580	0.041	0.595	0.059	0.600	0.093	0.592	0.065
110	0.992	0.101	1.036	0.104	1.038	0.097	1.021	0.099

Internal work rate's unit of measure is W kg^−1^; pedaling rate is in RPM; external work rate is presented in W and also in mass-specific units (i. e. W kg^−1^) in order to facilitate comparison with the associated internal work rates. Standard deviations for mass-specific external work rates of 50, 100, and 100 W were 0.08, 0.17, and 0.25, respectively.

**Figure 5:**
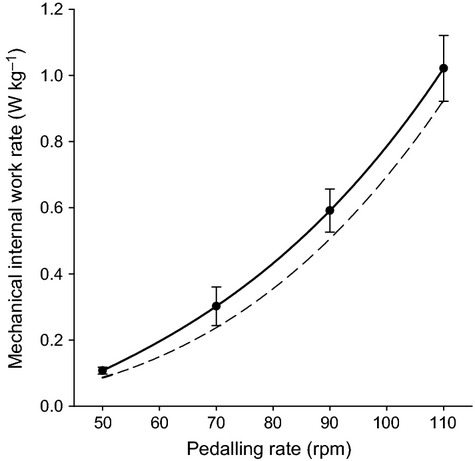
Mechanical internal work rate is shown against pedaling rate for the four pedaling rates considered (i.e., 50, 70, 90, and 110 revolutions per minute, rpm). Each filled circle represents the average mechanical internal work from ten participants for the corresponding pedaling rate; error bars represent standard deviation. The dashed line represents values calculated from Minetti (2011), and is shown as a means of comparison.

Figure 5 shows that a power function closely fits the experimental data and the obtained exponent is compatible with the modeling prediction [i.e., 3, (Minetti et al. 2001)]. The dashed curve in the figure, falling within 1 SD distance from the experimental results, represents the second term at the numerator of the ratio in eq. (3) [P_int_ (W kg^−1^) = 0.00000069 PR (RPM)^3^]. The close resemblance between the curve previously obtained (Minetti 2011) and the present one allows to confidently use eq. (4) to predict the metabolic power necessary to pedal on a cycle ergometer at a given external power and pedaling rate.

## Discussion

This study shows that that prediction of 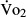 during exercise on the cycle ergometer through the ACSM equation can be improved by considering PR as a determinant of 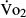. The new equation proposed here considers external work rate, individual's body mass and PR; it is particularly accurate at steady state, sub-maximal levels of exercise, and its application for exercise prescription is straightforward.

Several authors acknowledged the relevance of mechanical internal work in determining 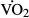 during cycling exercise [e.g., (Kautz and Neptune 2002; Minetti 2011)]. Equations and models that include mechanical internal work as a variable for the prediction of cycling 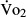 are often mathematically cumbersome. Presumably, this is the reason for which the ACSM recommends the use of a simpler equation, considering only an individual's body mass and mechanical external power, at the cost of reduced prediction accuracy. Equation MWPR, which only adds PR to the list of independent variables used by the ACSM equation, improved 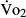 prediction, as shown by comparison with 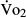 values measured in our participants. This improvement was due to a better 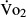 prediction starting from unloaded cycling and across the range of the 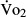 values measured. The limits of agreement in the prediction of 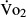 remained relatively large when using the MWPR equation, yet the limits of agreement obtained when using the ACSM equation were always greater. In particular, within-participant variance was more than three times greater when predicting 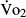 with the ACSM than with the MWPR equation. Depending on the conditions in which these equations are used, the magnitude of this variance may need to be considered, as it may have important physiological implications.

Although the MWPR and the ACSM equations are not intended for prediction of 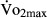, it is worth noticing that 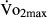 values predicted by the MWPR equation and those predicted by the ACSM equation were similar and that, overall, each of these equations slightly overestimated 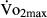 for the participants in this study. This similarity between the two equations, together with the improved accuracy of the MWPR equation in predicting submaximal 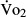 values, supports the use of the MWPR equation in a broader context, although its validation in the prediction of 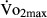 values across a larger population is required. While the bias was small, the observed limits of agreement could lead to an overestimation of 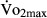, and may have clinically and physiologically relevant implications; this study was not sufficiently powered to detect these physiological differences.

One of the most supportive outcomes of this study was the accuracy in the prediction of 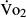 values reported in the literature (Gaesser and Brooks 1975; Seabury et al. 1977; Hagberg et al. 1981; Coast and Welch 1985; Francescato et al. 1995; Belli and Hintzy 2002). This part of the study allowed considering data from more than 50 participants, recorded in a variety of laboratories and conditions, and over almost thirty years, a series of factors that potentially challenged the prediction accuracy of 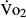 values. Despite the large variability in the data considered, the MWPR equation was able to predict 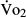 values that were close to the corresponding reported values. The agreement between 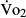 values predicted by the MWPR equation and 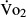 values reported in the literature was strong, especially for 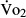 values up to 40 mlO_2_ kg^−1^ min^−1^. For greater 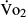 values some of the data points predicted by our equation were far from the identity line; the model presented here considers a fixed mechanical efficiency value, while it is possible that different values are associated to higher work rates. Also, it is possible that braking power dissimilarities between different types of ergometers has affected the variability in the prediction of the results reported in the literature.

We also studied the mechanics of exercise on the cycle ergometer, in order to explore the potential reasons for the MWPR equation's accuracy. The rationale used to propose a refinement of the ACSM equation is based on the concept that pedaling at different rates, independently from the external power produced, involves different amounts of the so-called ‘mechanical internal work’, that is, the work referring to reciprocal limbs' movement and to any friction, internal to our body, that needs to be overcome. For any mechanical external work rate level considered in this study, mechanical internal work rate showed a 10-fold increase between pedaling rates of 50 and 110 revolutions per minute, a result in agreement with previously reported values (Hansen et al. 2004). As metabolic power is strongly dependent on PR at any external work rate level on the cycle ergometer, it appears that the mechanical internal work rate at 110 revolutions per minute may become the greatest determinant of overall metabolic power at low external work rates. For example, our results suggest that when cycling at 50 W with a PR of 110 revolutions per minute, mechanical internal work rate would be approximately 75 W for an individual of 75 kg, which corresponds to 150% of the external work rate; taking into account the PR for 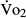 determination is important all the more as the work rate is low and the PR is high. In contrast, at elevated external power levels, the proportion of metabolic power associated with mechanical internal work rate becomes relatively smaller. These values *per se* may be confounding, and appropriate conclusions regarding optimal pedaling rates for efficient exercise can only be drawn in view of the intrinsic biomechanical characteristics of skeletal muscle physiology (Kautz and Neptune 2002; Emanuele and Denoth 2012). Particularly, muscular contraction efficiency shows a maximum (0.25–0.28) at 15–29% of its maximum shortening speed (Hill 1964; Woledge et al. 1985); below and above this speed (directly proportional to the pedaling rate) muscle needs more than fourfold metabolic power to generate the same external mechanical power (Minetti, in preparation). Thus, at present, the main determinants of the metabolic expenditure increase at high pedaling rates (for a given WR) are still debated. Candidates include: (1) the mechanical internal work, either the kinematic or the friction components (Minetti 2011), or a combination of both, (2) the additional mechanical external work deriving from the 3D trajectory of the body center of mass that, differently from the common belief, does not move at a constant speed and a constant height from the ground while cycling (Minetti 2011), and (3) the efficiency of muscular contraction that, if always operating at speed greater than the optimum one, could explain the metabolic power needed.

The results presented in this study are limited to a relatively small population of male participants, and the validity of the MWPR equation needs to be confirmed in a wider sample including female participants, and a variety of populations from different age and patients’ groups, physique, health and training status among other characteristics. The agreement discussed above, between values predicted by the MWPR equation and those reported in the literature, is certainly supportive at this stage.

In conclusion, this study presents a novel equation for the prediction of submaximal 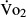 during exercise on the cycle ergometer. This equation increases the 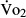 prediction accuracy of the ACSM equation, at the only cost of considering pedaling rate as an additional determinant.
